# Public knowledge and practices toward safe disposal of unused and expired medications in Palestine: a cross-sectional study

**DOI:** 10.3389/fpubh.2026.1944632

**Published:** 2026-09-01

**Authors:** Anas K. Assi, Alhareth M. Amro, Salahaldeen Deeb, Habeeb H. Awwad, Malak K. Assi, Ahmad K. Assi, Samer Amayra, Ali Nassr, Omar Jehad Arman, Abdalqader Alwohoush, Ahmed Zaid, Mohammad Amouri, Riman A. Sultan

**Affiliations:** 1 Faculty of Medicine, Al-Quds University, Jerusalem, Palestine; 2 Faculty of Medicine, An Najah National University, Nablus, Palestine; 3 Faculty of Medicine, Arab American University, Ramallah, Palestine; 4 Emergency Department, Palestine Medical Complex, Jerusalem, Palestine

**Keywords:** attitudes, environmental health, knowledge, medication disposal, Palestine, pharmaceutical waste, practices, public health

## Abstract

**Background:**

Improper disposal of unused and expired medications poses significant health and environmental risks worldwide, especially in resource-limited and conflict-affected settings where pharmaceutical waste management infrastructure is lacking and antimicrobial resistance (AMR) is rising. Population-level data from fragile healthcare systems in the Middle East are scarce. This study assessed knowledge, attitudes, and practices (KAP) related to medication disposal among Palestinian adults in a setting without formal take-back programs or national disposal guidelines, and analyzed behavioral determinants to inform pragmatic AMR prevention and waste management strategies.

**Methods:**

A cross-sectional study was conducted using an online survey. A total of 402 non-healthcare Palestinian adults (≥18 years) were recruited via non-probability snowball sampling. A structured questionnaire was administered online. While this approach enabled wide reach; however, it may have limited representativeness of older and less digitally connected individuals. We utilized multivariable logistic regression to identify predictors of behavior based on HBM constructs. To strengthen inferential validity, causal mediation analysis was performed to assess whether perceived barriers mediated the relationship between risk perception and disposal practices. Internal consistency was assessed with Cronbach’s *α*, and data were analyzed using SPSS v25 with a *p* < 0.05 considered significant.

**Results:**

Respondents were predominantly female (67.4%) with mean age 27.2 ± 9.6 years. Specific environmental knowledge was limited: 40.8% knew flushing contaminates groundwater and 29.4% linked improper disposal to antibiotic resistance. Although 93% supported professional guidance and public awareness, unsafe practices predominated: 77.4% discarded expired medications in household garbage and only 9.7% returned them to pharmacies. Multivariable analysis showed perceived risk was the strongest positive predictor of safe disposal, while perceived barriers significantly reduced odds of safe behavior. Mediation analysis found no significant indirect effect of risk via barriers, indicating independent pathways. Weighted sensitivity analyses confirmed the robustness of the risk–behavior association.

**Conclusion:**

Positive attitudes coexist with inadequate disposal practices in Palestine. Structural barriers, not lack of risk awareness alone, sustain unsafe behaviors. Effective policy must combine risk communication with removal of logistical obstacles—most feasibly through accessible pharmacy-based take-back programs and supportive operational policies—to translate awareness into safer disposal and reduce environmental risks.

## Introduction

The accumulation of unused and expired medications in households has emerged as a growing public health and environmental concern worldwide. Recent household surveys still report large volumes of unused and expired pharmaceuticals remaining in homes, representing an ongoing source of pharmaceutical waste and increasing the chance of improper disposal (1, 2). Community-level surveys from Saudi Arabia and other low- and middle-income settings have reported that household waste disposal is the most commonly used method for unwanted medication disposal, and only a minority of individuals use appropriate disposal pathways such as pharmacy-based collection programs (1, 3, 4). Advances in medical care have increased medication use and, consequently, pharmaceutical waste streams (5). According to the World Health Organization (WHO), more than half of all pharmaceuticals are prescribed, delivered, or marketed inappropriately, and half of all patients do not take their prescription properly. These practices create pharmaceutical waste and related health risks (6). Unused drugs often result from therapy changes, side effects, or patient non-adherence and are frequently discarded improperly, contributing to environmental pollution and potential health hazards (1, 5).

Active pharmaceutical ingredients released into the environment through unsafe disposal can contaminate water and soil, promote antibiotic resistance, and threaten ecosystems (7, 8). The Organization for Economic Co-operation and Development (OECD) emphasizes that expired and unused pharmaceuticals should be managed through safe disposal systems to minimize risks to human health and environmental contamination (8). While many high-income countries and certain middle-income countries have implemented medicine take-back programs, recent research from Iran, Saudi Arabia, Ethiopia, Nepal, and South Africa indicates that domestic waste remains the predominant disposal method in these specific settings due to a lack of, or absence of, coordinated collection systems (1, 8, 9).

The Knowledge, Attitudes, and Practices (KAP) framework is commonly used to assess public awareness and behaviors regarding health issues, including medication disposal (3, 10, 11). The Health Belief Model (HBM) further explains how individuals’ perceptions of risks, benefits, and barriers influence their health-related behaviors. In this study, HBM constructs were incorporated into the attitude items to assess perceived risks, barriers, and cues to action related to safe disposal (12). In Palestine, a fragile and conflict-affected setting with limited healthcare infrastructure, there is currently no formal take-back program or national guideline for the disposal of household medications. This gap is particularly concerning given rising rates of antimicrobial resistance in the region and the absence of environmental monitoring for pharmaceutical pollutants in water and soil systems. Previous studies in Palestine were mainly on the disposal habits of community members and health care personnel, not on the general population (9, 13), leaving a critical gap in population-level data regarding the general public’s knowledge, attitudes, and practices. Leaving a critical gap in population-level data regarding the general public’s knowledge, attitudes, and practices. Understanding public disposal behaviors in such settings is essential for developing feasible, context-appropriate interventions that can simultaneously address environmental contamination and AMR prevention without requiring capital-intensive infrastructure.

To address this gap, the present study aimed to assess the KAP regarding the disposal of unused and expired medications among Palestinian adults and to identify behavioral determinants. Importantly, this study employs causal mediation analysis to distinguish between independent motivators (risk perception) and structural barriers (inconvenience, lack of information)—a methodological approach that provides actionable insights for policy design in resource-limited settings. The findings contribute to the limited evidence base on pharmaceutical waste management in fragile healthcare systems and offer a scalable implementation framework adaptable to similar low- and middle-income countries facing concurrent challenges of inadequate waste infrastructure and rising antimicrobial resistance.

## Methods

### Study design and setting

A community-based cross-sectional survey was conducted from March to August 2025 to assess knowledge, attitudes, and practices regarding the disposal of unused and expired medications among the general population in Palestine. Data were collected through an online self-administered questionnaire distributed via social media platforms (e.g., Facebook and WhatsApp) using a snowball non-probability sampling technique. This approach enabled rapid and broad dissemination but may have introduced selection bias, as participation was limited to individuals with internet access and active social networks. Consequently, the sample may overrepresent younger, more educated, and urban populations, limiting generalizability to all Palestinian adults.

### Participants

Eligible participants were Palestinian residents aged 18 years or older. To ensure that results reflected the general public’s awareness rather than professional or academic knowledge, healthcare professionals and students in medical or health-related fields were excluded. This approach was adopted to minimize bias and better capture the population-level understanding of medication disposal. While this exclusion may limit comparability with studies involving healthcare workers, it enhances the internal validity of findings related to the general community. Participation was voluntary, anonymous, and based on informed consent implied through survey completion.

### Sample size

The minimum required sample size was estimated using the Raosoft^®^ online calculator, assuming a 95% confidence level, 5% margin of error, and 50% response distribution, which yielded a minimum of 377 participants. To enhance precision and account for incomplete responses, data collection continued until 402 valid responses were obtained. Although this approach ensures adequate power for estimating overall population parameters, the sample size calculation was not stratified by subgroups. Therefore, analyses comparing demographic groups (e.g., gender, education, or income) were considered exploratory and interpreted with caution, as some categories contained relatively small numbers of participants.

### Study instrument

The survey instrument was adapted from previously published and validated KAP questionnaires on medication disposal by Mohammed et al. (7) in Iraq. The original English version was translated into Arabic by two bilingual public health researchers, then back-translated into English by an independent translator who was blinded to the original text. Discrepancies were resolved through consensus discussion to ensure conceptual equivalence. It comprised 36 items organized into four sections: (1) socio-demographic and household information (age, gender, education, income, family size, etc.); (2) knowledge items (8 true/false questions); (3) attitude statements (10 statements rated on a five-point Likert scale from “strongly disagree” to “strongly agree”); and (4) practice questions (6 items on disposal behaviors and frequency). Minor cultural adjustments were made to improve contextual relevance, such as updating examples of commonly used medications and modifying terminology to align with local healthcare settings. The Arabic version was pilot-tested among 25 adults from the target population to assess clarity and comprehensibility. Feedback from this pilot resulted in minor wording refinements. The final instrument was reviewed and approved by a panel of public health experts before data collection.

Knowledge items were scored dichotomously (1 point for correct answers and 0 points for incorrect answers), whereas attitude items were scored using a 5-point Likert scale. The lower Cronbach’s alpha observed for the knowledge domain was expected, as dichotomous factual items generally exhibit lower internal consistency than Likert-scale attitude measures. The first item in the practice section, “Do you have unused medications at home?” functions as a screening question for prevalence, not a measure of a disposal practice itself. A panel of public health experts reviewed the questionnaire for clarity and relevance. Internal consistency was assessed via Cronbach’s alpha: the knowledge subscale yielded *α* ≈ 0.66 and the attitude subscale *α* ≈ 0.80, indicating acceptable reliability in this sample.

### Data collection

An online questionnaire (Google Forms) was developed and distributed according to the above plan. To prevent duplicate submissions, the “Limit to 1 response” setting was enabled, requiring participants to be signed into a Google account. Respondents were informed that the study was for research purposes only, that their responses would remain confidential, and that they could withdraw at any time. The survey included only closed-ended questions (multiple-choice or Likert scale) and took approximately 7–10 min to complete. Data were automatically recorded into a secure database; completeness checks were performed, and any partially completed forms were excluded. Incomplete submissions were excluded if any of the main sections (knowledge, attitudes, or practices) were left unanswered or if more than 10% of total items were missing.

### Ethical considerations

All procedures performed in this study involving human participants complied with the institutional and/or national research committee ethical standards and the 1964 Helsinki declaration and subsequent amendments or equivalent ethical standards. The study was designed and conducted in accordance with the ethical principles established by Al-Quds University. Therefore, ethical approval was obtained from the Institutional Review Board Committee, Al-Quds University. All participants provided digital informed consent before beginning the online survey. The first page of the survey presented a detailed information statement describing the study’s purpose, confidentiality, and voluntary participation. Participants indicated their consent by selecting the “I agree to participate” checkbox before proceeding to the questionnaire. Data were anonymized, stored securely, and accessible only to authorized researchers. Participants were assured they could withdraw from the study at any time without repercussions.

### Statistical analysis

Data were exported to SPSS Statistics v25 (IBM Corp.) for analysis. Descriptive statistics summarized participant characteristics and KAP responses (means with standard deviations for continuous variables; frequencies and percentages for categorical variables). Between-group comparisons were performed using independent-samples *t*-tests for continuous outcomes and chi-square tests for categorical variables. Differences in participants’ attitudes by gender were assessed using the Mann–Whitney *U* test, as the attitude items were measured on an ordinal Likert scale. Statistical significance was defined as *p* < 0.05. Given multiple comparisons, findings may be subject to inflated Type I error rates. Results should therefore be interpreted as exploratory. All analyses followed standard methods for cross-sectional KAP surveys.

To strengthen the analytical framework, the Health Belief Model (HBM) was operationalized by creating composite scores for three key domains: Perceived Risk, Cues to Action, and Perceived Barriers. The ‘Perceived Risk’ score was derived by averaging knowledge items regarding environmental harm, groundwater contamination, and antibiotic resistance with the attitude item on risks to children. ‘Cues to Action’ aggregated support for professional advice, public awareness campaigns, and financial incentives. ‘Perceived Barriers’ combined the reported lack of information with the perceived inconvenience of pharmacy take-back (reverse-coded). These composite HBM scores were entered as continuous predictors in a multivariable binary logistic regression model alongside sociodemographic variables (age, gender, education, and income) to determine their independent association with safe disposal practices (defined as returning medications to a pharmacy or proper incineration). Multicollinearity was assessed using the variance inflation factor (VIF), and no significant collinearity was observed (all VIF < 2). Model fit was evaluated using Nagelkerke’s *R*^2^ and the Hosmer–Lemeshow goodness-of-fit test. Predictive performance was assessed using the Area Under the Receiver Operating Characteristic Curve (AUC). Finally, to assess the robustness of the findings against potential sampling bias, a sensitivity analysis was performed using post-stratification weighting (raking). Weights were generated to align the sample distribution with general Palestinian population estimates for age, gender, and education level.

## Results

The demographic characteristics of the 402 survey respondents are detailed in (Table 1). The sample was predominantly female (67.4%), young (mean age = 27.2 ± 9.6 years), and highly educated (81.8% holding a university degree). Monthly income was predominantly within the average range (60.7%), while 30.3% reported low income. Most participants were single (64.4%) and students (44.8%). Notably, only 16.9% of participants reported having previously received information on safe medication disposal, and 45.0% had a healthcare professional in the household. The average household size was 6.4 members (SD = 2.3).

**Table 1:** Descriptive statistics of the participants.

Parameter	Category	*N* (%) or mean ± SD
Gender	Male	131 (32.6%)
	Female	271 (67.4%)
Marital status	Single	259 (64.4%)
	Married	136 (33.8%)
	Other	7 (1.7%)
Education level	University	329 (81.8%)
	Postgraduate studies	39 (9.7%)
	Secondary or less	34 (8.5%)
Occupation	Student	180 (44.8%)
	Employed	157 (39.1%)
	Other/not employed	65 (16.2%)
Monthly income	Low	122 (30.3%)
	Average	244 (60.7%)
	High	36 (9.0%)
Healthcare professional at home	Yes	181 (45.0%)
	No	221 (55.0%)
Received disposal information	Yes	68 (16.9%)
	No	334 (83.1%)
Age (years)	Mean ± SD	27.2 ± 9.6
Family members in home	Mean ± SD	6.4 ± 2.3

Participants’ knowledge revealed a gap between general awareness and specific risks (Table 2). While a majority (73.4%) recognized that improper disposal harms the environment, specific knowledge was low. For instance, only 40.8% knew that flushing medications contaminates groundwater, and only 29.4% understood the link between improper antibiotic disposal and antimicrobial resistance. Knowledge of correct disposal methods was also poor, with only 13.7% identifying incineration as an environmentally sound method.

**Table 2:** Frequency of correct and incorrect answers for knowledge questions.

Knowledge item	Correct *N* (%)	Incorrect *N* (%)
Improper disposal of unused/expired medications harms the environment.	295 (73.4%)	107 (26.6%)
Medications can reach groundwater if thrown into the toilet/sink.	164 (40.8%)	238 (59.2%)
Disposing solid medications (pills/capsules) in garbage is acceptable.	132 (32.8%)	270 (67.2%)
Unsafe disposal of antibiotics (e.g., amoxicillin) leads to antibiotic resistance.	118 (29.4%)	284 (70.6%)
Incineration is an environmentally sound way to dispose unwanted medications.	55 (13.7%)	347 (86.3%)
Disposing needles and syringes in the garbage is acceptable.	213 (53.0%)	189 (47.0%)
Disposing asthma inhalers in the garbage is acceptable.	155 (38.6%)	247 (61.4%)
Disposing creams and ointments in the garbage is acceptable.	103 (25.6%)	299 (74.4%)

Attitudes toward safe disposal were generally positive (Table 3). A large majority felt a personal responsibility to protect their families (80.6%) and strongly supported professional guidance (93.2%) and public awareness programs (93.0%). However, attitudes were ambivalent regarding the role of pharmacies; fewer than half (46.8%) agreed on the important role of community pharmacists, and only 55.9% found returning medications to pharmacies convenient.

**Table 3:** Attitudes of the participants toward disposal of unused/expired medications.

Attitude item	Strongly disagree *N* (%)	Disagree *N* (%)	Neutral *N* (%)	Agree *N* (%)	Strongly agree *N* (%)	Median (IQR)
It is my responsibility to protect my family from harmful exposure to unused/expired medications.	7 (1.7%)	9 (2.2%)	62 (15.4%)	144 (35.8%)	180 (44.8%)	4 (1)
I am willing to donate my unused medications before they expire to reduce waste.	16 (4.0%)	25 (6.2%)	46 (11.4%)	177 (44.0%)	138 (34.3%)	4 (1)
A financial incentive to return unused medications would motivate me to do so.	9 (2.2%)	38 (9.5%)	91 (22.6%)	167 (41.5%)	97 (24.1%)	4 (1)
Doctors, pharmacists, and health professionals should provide advice on safe disposal of medications.	4 (1.0%)	3 (0.7%)	20 (5.0%)	134 (33.3%)	241 (59.9%)	5 (1)
Awareness programs on how to dispose unused/expired medications should be initiated.	2 (0.5%)	3 (0.7%)	19 (4.7%)	146 (36.3%)	232 (57.7%)	5 (1)
There should be public awareness programs about the harmful effects of improper disposal.	3 (0.7%)	6 (1.5%)	14 (3.5%)	162 (40.3%)	217 (54.0%)	5 (1)
Community pharmacists have an important role in preventing improper disposal of medications.	28 (7.0%)	53 (13.2%)	133 (33.1%)	170 (42.3%)	18 (4.5%)	3 (1)
There is a lack of sufficient information on safe disposal of unused/expired medications.	3 (0.7%)	8 (2.0%)	83 (20.6%)	212 (52.7%)	96 (23.9%)	4 (0)
Children are at high risk from unused/expired medications at home.	6 (1.5%)	4 (1.0%)	59 (14.7%)	165 (41.0%)	168 (41.8%)	4 (1)
It is convenient for me to take unused/expired medications to the pharmacy for disposal.	16 (4.0%)	64 (15.9%)	97 (24.1%)	136 (33.8%)	89 (22.1%)	4 (1)

Reported practices were often unsafe (Table 4). 68.4% of respondents had unused medicines at home, and 76.8% disposed of medicines when they changed color or appearance. Expiry-date checking was common: 62.2% checked always or most of the time (38.3% always, 23.9% usually). Most expired medications (77.4%) were discarded in household garbage; only 9.7% were returned to a pharmacy, 5.5% incinerated, and 4.5% flushed. For unused but unexpired medications, 40.3% were thrown in the garbage, 13.9% given to others, and 9.7% returned to pharmacies.

**Table 4:** Practice of the participants toward disposal of unused/expired medications.

Practice item	Subcategory	*N* (%)
Do you have unused medications at home?	Yes	275 (68.4%)
	No	127 (31.6%)
I dispose medications when their color changes or they look bad.	Yes	309 (76.8%)
	No	93 (23.2%)
How often do you check expiration dates?	Always	154 (38.3%)
	Most of the time	96 (23.9%)
	Sometimes	70 (17.4%)
	Rarely	58 (14.4%)
	Never	24 (6.0%)
How do you dispose expired medications?	Home garbage	311 (77.4%)
	Return to pharmacy	39 (9.7%)
	Incinerate	22 (5.5%)
	Flush into toilet/sink	18 (4.5%)
	Keep at home	1 (0.2%)
	Other	11 (2.7%)
Other disposal methods (for expired medications)	Landfill	31 (7.7%)
	Burning	10 (2.5%)
	Deep burial	7 (1.7%)
	Returned or passed leftover medicines to others or to a health facility	12 (3.0%)
	I do not know	10 (2.5%)
What do you do with unused medications?	Keep until expire	121 (30.1%)
	Dispose in garbage	162 (40.3%)
	Give to friends/relatives	56 (13.9%)
	Return to pharmacy	39 (9.7%)
	Flush into toilet/sink	14 (3.5%)
	Other	10 (2.5%)

“Burning” refers to the open destruction of medicines by fire in household or open settings (not controlled incineration). “Deep burial” refers to burying medicines in a pit or trench covered with soil.

To explore potential variations in knowledge, attitudes, and practices across demographic subgroups, comparisons were conducted by gender, education level, age, and income. However, these subgroup analyses are exploratory in nature, as several demographic categories contained relatively small numbers of participants, which limits statistical power and generalizability. Therefore, these findings should be interpreted cautiously due to limited group sizes and potential sample bias.

Comparison of knowledge scores by gender showed that most items did not differ significantly between males and females (*p* > 0.05). However, women performed significantly better on two items. A higher proportion of female respondents correctly identified that disposing of solid medications in household garbage is unacceptable (60.6% vs. 39.4% for males, *p* = 0.042). Similarly, females were more likely to answer correctly regarding the disposal of inhalers (73.5% vs. 26.5% for males, *p* = 0.038) (Supplementary Table 1).

Analysis of attitudes by gender revealed three items with statistically significant differences (Supplementary Table 2). Female participants rated the need for disposal-awareness programs more highly than males (median 5, IQR 1 vs. median 4, IQR 1; *p* = 0.003) and expressed stronger support for public awareness initiatives (median 5, IQR 1 vs. median 4, IQR 1; *p* < 0.001). In addition, analysis of the response distributions showed women were more likely to agree that there is insufficient information available on safe disposal (median 4, IQR 1 vs. median 4, IQR 1; *p* = 0.012).

Knowledge differences by education were minimal (Supplementary Table 3). The only significant variation was for the item on environmental harm: correct responses were 61.8% for respondents with secondary education or less, 76.0% for university graduates, and 61.5% for postgraduates (*p* = 0.043) (Supplementary Table 3). Other knowledge items showed no significant differences by education level (all *p* > 0.05). Because the number of respondents with lower education was relatively small, results for this subgroup should be interpreted with caution (see Table 5).

**Table 5:** Multivariable logistic regression predicting safe medication disposal behaviors (*N* = 402).

Predictor	Adjusted odds ratio (AOR)	95% confidence interval (CI)	*p*-value
Sociodemographic factors			
Age	0.99	0.95–1.03	0.729
Gender (female vs male)	1.03	0.50–2.22	0.936
Education level	0.69	0.35–1.45	0.309
Monthly income	0.51	0.27–0.93	0.031*
Health belief model constructs			
Perceived risk	37.43	7.99–197.32	<0.001***
Perceived barriers	0.47	0.26–0.83	0.011*
Cues to action	0.63	0.35–1.15	0.123
Model performance statistics			
Nagelkerke *R*^2^	0.174		
Hosmer–Lemeshow test	*X*^2^ = 5.33, *p* = 0.722		
Area under the curve (AUC)	0.782		

*p* < 0.05 indicates a statistically significant association. Bold values indicate statistically significant associations (*p* < 0.05).

Analysis of knowledge by age revealed significant differences for two items. Participants who incorrectly identified the acceptability of discarding solid medications in household garbage were older on average than those who answered correctly (mean age 28.1 vs. 25.4 years, *p* = 0.008). Similarly, respondents who answered the syringes/needles item incorrectly had a higher mean age compared with those who answered correctly (28.4 vs. 26.1 years, *p* = 0.016) (Supplementary Table 4).

Knowledge differences across income groups were generally limited. The only significant association was observed for the item on groundwater contamination, where correct responses increased with income level (*p* = 0.026). Specifically, 33.6% of respondents in the low-income group answered correctly, compared with 41.8% in the average-income group and 58.3% in the high-income group (Supplementary Table 5).

The multivariable logistic regression model (Nagelkerke *R*^2^ = 0.174) identified specific HBM constructs as critical drivers of disposal behavior. Perceived risk emerged as the strongest predictor; participants with higher risk perception were substantially more likely to practice safe disposal (AOR = 37.43, 95% CI: 7.99–197.32, *p* < 0.001). The wide confidence interval observed for this estimate is attributable to the relatively low frequency of safe disposal events within the sample, which inflates the standard error of the logistic regression model, though the lower bound remains robustly positive. Conversely, perceived barriers significantly hindered safe practices; for every unit increase in reported barriers (e.g., inconvenience or lack of information), the odds of safe disposal decreased by approximately 53% (AOR = 0.47, 95% CI: 0.26–0.83, *p* = 0.011). Cues to action, such as support for awareness programs and professional advice, did not show a statistically significant association with actual behavior in this model (AOR = 0.63, *p* = 0.123). Among demographic variables, only monthly income showed a significant association in the adjusted model (AOR = 0.51, *p* = 0.031), while age, gender, and education were not significant predictors. The model demonstrated good discriminatory power, with an Area Under the Curve (AUC) of 0.782, indicating a high probability of correctly classifying disposal behavior. Furthermore, the Hosmer–Lemeshow test indicated good model calibration (*Χ*^2^ = 5.33, *p* = 0.722), suggesting no significant deviation between observed and predicted values.

To further elucidate the pathways between HBM constructs and disposal behavior, a causal mediation analysis was conducted. The analysis tested whether Perceived Barriers mediated the relationship between perceived risk and safe disposal. The results revealed a significant Average Direct Effect (ADE) of perceived risk on disposal behavior (estimate = 0.24, 95% CI: 0.14–0.35, *p* < 0.001), confirming that higher risk awareness directly increases the probability of safe disposal. However, the Average Causal Mediation Effect (ACME) was not statistically significant (estimate = 0.001, *p* = 0.896), indicating that perceived risk does not influence behavior by lowering perceived barriers. Instead, risk sensitivity analysis.

To ensure that the study’s findings were not driven by the demographic skew toward younger and highly educated participants, a weighted logistic regression analysis was conducted. After adjusting for age, gender, and education to match population estimates, perceived risk remained a statistically significant predictor of safe disposal (*p* = 0.006). Perceived barriers exhibited a consistent negative coefficient (Estimate = −0.76) comparable to the primary model, although the association became marginally significant (*p* = 0.08) due to the increased variance inherent in weighted analyses. This stability in effect sizes suggests that the identified associations are robust and not solely attributable to sampling bias.

## Discussion

This study provides a comprehensive assessment of public KAP regarding medication disposal in Palestine, revealing both encouraging attitudes and critical gaps in knowledge and practice. For example, most respondents recognized that improper disposal can harm the environment, but far fewer knew correct disposal methods. Overall, many technical questions were answered incorrectly, indicating gaps in detailed knowledge. By contrast, knowledge of general risks (e.g., environmental harm) was relatively high. This pattern matches global findings: a recent systematic review of household pharmaceutical waste disposal concluded that knowledge is often “limited to inadequate,” even when attitudes are favorable (14). Initial cross-sectional research in low- and middle-income settings has found similar knowledge gaps. Kahsay et al. (4) reported that while many participants in Ethiopia knew that improper disposal could be harmful, there was limited knowledge of safe disposal procedures and home disposal was often regarded as an acceptable alternative. Similarly, Mahlaba et al. (6) in South Africa showed that patients were often unaware of the appropriate disposal methods and there were many misconceptions regarding the safety of normal disposal in the household.

These results are quite similar to ours, which indicated a rather high level of awareness of environmental hazards but a poor understanding of specific safe disposal methods. Some populations show higher awareness but these tend to be organized health-survey settings (11). Differences in participant characteristics, questionnaire design, and past educational exposure may explain differences in reported knowledge levels. Increased access to health information in communities or urban centers might be an indication of heightened awareness, but online polls conducted in areas with limited public health campaigns may show persistent gaps in knowledge. Findings converged across contexts, which may reflect a greater public knowledge of health risks of environmental pollution through health messaging, while technical aspects of pharmaceutical waste treatment require specialized instruction.

In Palestine the gap may be widened by the absence of national disposal norms and few public campaigns. Overall, our data suggest that Palestinians, like many groups worldwide, know that improper disposal is harmful but lack detailed knowledge of how to dispose safely (3). Recent evidence from Iran provides evidence of this tendency. Iranian families knew of the dangers of prescription waste, but the behaviors of improper disposal were still common, showing that broad awareness does not necessarily result in appropriate disposal behaviours (1). The similarity of the Palestinian, Iranian and other Middle Eastern populations may be due to common health system challenges such as poor public awareness campaigns, lack of organized collection programmes for household pharmaceutical waste and limited integration of medication disposal counselling into routine healthcare services. In many low- and middle-income settings, take-back systems lack the regulatory frameworks and pharmacy networks of high-income nations, and depend on human responsibility without practical disposal options.

The attitudes of our respondents were generally positive and proactive. Over 80% felt personal responsibility for protecting their family from medication hazards, and more than 90% supported professional guidance and public awareness campaigns. Many (78%) were even willing to donate unused medications to reduce waste. The findings are in line with the initial studies in Nepal and Ethiopia. Education on how to dispose of medicines and supervision by experts are favorable in Nepalese communities, but dangerous practices are still common (11). Likewise in Ethiopia, a study conducted by Kahsay et al. (4) revealed that people had a positive attitude towards appropriate pharmaceutical waste management, indicating that there was a willingness to change disposal practices despite the absence of infrastructure. A recent study in Saudi Arabia (Khan et al.) found that awareness and perceived environmental hazards affect public perception of safe disposal. They emphasized the need for education and practical ways of disposal (2). Importantly, systematic reviews.

note that majorities almost universally favor take-back initiatives and education (14). One nuance in our data is that only 55.9% of Palestinians agreed it is “convenient” to return unused meds to a pharmacy, and many (42.3%) were unsure that community pharmacists have an important role. This suggests a slight ambivalence about pharmacy-based solutions. In contrast, most international programs rely on pharmacies as collection points (5). Nonetheless, the high support for professional guidance and public campaigns indicates strong motivation to improve practices, provided accessible options exist (11). The generally favorable attitudes despite inadequate disposal practices may reflect a common public health gap, where individuals recognize concerns but cannot translate intentions into action without supportive systems. Similar attitude–practice discrepancies in Nepal, Ethiopia, and Saudi Arabia may reflect limited convenient disposal facilities rather than unwillingness among community members.

Despite positive attitudes, actual disposal practices were poor. Unsafe disposal practices predominated in our sample, and most respondents reported disposing of unwanted medications in household trash, with very few using pharmacies or other safe routes. These findings are consistent with the recent data from neighboring Middle Eastern countries. The inappropriate disposal of expired and unwanted drugs was common among the Iraqi population, and disposal in household garbage was the most prevalent (7). Mohammed and Al-Hamadani reported that Similarly, Althagafi et al. (1) observed that Saudi participants knew about the potential risks of drug disposal but disposed of drugs using risky methods. Mostafanejad et al. (8) observed that the disposal practices of Iranian families were mostly inappropriate and the disposal of the prescription waste is still a regional public health problem. These studies, as well as our results, suggest that the problem of improper disposal is not unique to Palestine, but rather a symptom of a broader regional gap between awareness and available disposal infrastructure. Even in higher-income settings, improper disposal remains common (5). These convergent findings highlight a global attitude–practice gap—people acknowledge the importance of safe disposal but continue unsafe methods (2, 14, 15).

In Palestine, Nairat et al. (9) found that community pharmacies were not prepared to manage pharmaceutical waste and concluded that the absence of planned means of disposal is not only a public issue but also a problem in the health care delivery system. Our findings add to existing data by showing that Palestinian families have no means of safe disposal of medications. Palestine currently has no official national medication take-back program or clear public guidelines on household disposal, and public health campaigns on this topic are limited. Without accessible disposal alternatives, many people default to convenient but unsafe methods. Cultural and economic factors may also play a role: for example, it was common in our sample to share unused medications with family or friends, reflecting social norms of resource sharing, but this practice can contribute to widespread misuse and complicate disposal efforts.

By operationalizing the Health Belief Model, our analysis provides a nuanced understanding of the disconnect between attitude and practice. The regression results highlight that Perceived Risk is a powerful motivator (AOR = 37.43), while Perceived Barriers significantly hinder safe practice (AOR = 0.47). Perceived barriers may be particularly salient in Palestine, where formal medication take-back infrastructure does not exist. Thus, there may be practical constraints facing residents that may limit behavior change despite high-risk perception. Crucially, our causal mediation analysis demonstrated that these two factors operate as independent, parallel determinants rather than a causal chain (ACME *p* = 0.896). This indicates that high risk perception drives the motivation to act (ADE *p* < 0.001), but it does not mitigate the structural barriers hindering the action. This distinction is vital for policymaking: it implies that public health campaigns focused solely on education (increasing risk awareness) will fail to maximize safe disposal because they do not address the physical obstacles. A high-awareness individual is still blocked by a lack of convenient drop-off points. Therefore, a successful national strategy must be dual-pronged: pairing educational initiatives directly with infrastructure improvements that lower physical barriers, such as pharmacy-based take-back schemes.

The similarity of our findings with recent original research from the Middle East, Africa and Asia demonstrates that the problem of inappropriate disposal of home medicines is a worldwide issue and not only related to a lack of awareness but also to a lack of infrastructure and policy implementation. Future efforts will thus need to combine community education with sustainable collection mechanisms that are compatible with local health care settings.

### Implications for policy and practice

These findings have important policy and practice implications, suggesting that a dual strategy involving infrastructure development and targeted education is necessary. First, the results strongly support implementing a formal medication take-back system in Palestine. Our respondents’ high willingness to return medicines (and donate them) suggests public cooperation, and the successful use of pharmacies as collection points elsewhere indicates a feasible model (5). In Palestine, no formal household medication disposal policy or program currently exists. Ministry of Health (MoH) data (albeit scarce) indicate growing pharmaceutical waste volumes, yet waste management guidelines focus on healthcare facilities, not community disposal (13). Policymakers should consider developing national guidelines, in alignment with WHO recommendations, including pilot take-back programs to assess the feasibility and optimal implementation strategy for sustainable pharmaceutical waste management (5). Funding and publicizing periodic take-back events, or establishing permanent drop-off boxes at pharmacies, would align with FDA and EMA guidelines that such programs are “best” disposal methods (5). Second, educational interventions are urgently needed. Even brief educational campaigns have been shown to improve awareness among pharmacists and the public. The fact that only 16.9% of our sample had received any prior information on safe disposal underlines a huge knowledge gap. Health authorities and Non-Governmental Organizations (NGOs) should run media campaigns (TV, radio, and social media) and school programs to explain that “flushing” and “throwing” are harmful, and to publicize any new disposal services. Pharmacy practice should be leveraged: nearly all participants agreed that pharmacists/doctors should give advice. Thus, training community pharmacists to counsel patients on disposal (as many now counsel on dosages) could be impactful. Third, these efforts would serve broader environmental and public health goals. Improper disposal contributes to pharmaceutical contamination of water and soil and may accelerate antimicrobial resistance (15, 16). For example, antibiotics disposed in wastewaters can select resistant bacteria in the environment. Ensuring safe disposal is therefore part of global sustainable development and Antimicrobial Resistance (AMR) containment strategies. This is consistent with recommendations from international reviews and the WHO that multi-pronged strategies are needed for this issue (5).

### Strengths and limitations

A major strength is the relatively large sample (*N* = 402) and use of a structured, validated questionnaire adapted from a recent Iraqi study (8). This study considered one of the first large-scale surveys on household medication disposal in Palestine. The study adheres to STROBE reporting standards, ensuring transparency. Analytical rigor is demonstrated by assessing reliability (Cronbach’s alpha) for our scales, enhancing the credibility of our KAP measurements. The survey covered knowledge, attitudes, and practices comprehensively and the questionnaire was reviewed by a panel of public health and pharmacy experts to ensure clarity, content validity, and contextual relevance. Although some knowledge items (e.g., awareness of environmental harm or child-related risks) may appear to overlap with attitudinal constructs, they were retained under the knowledge domain based on expert consensus rather than subjective opinion.

Online administration allowed rapid data collection with anonymity, likely reducing social desirability bias. Compared to other KAP surveys, our sample has some diversity. Our study’s strengths lie in its use of a previously tested instrument and formal statistical analysis of demographic correlates; few KAP surveys (like ours and Iraq’s) examined how gender, age, education or income affect responses. Indeed, we found that females scored better on some knowledge items and felt more strongly about awareness programs, and that higher education/income was modestly linked to environmental awareness. These subgroup findings enrich the literature on how socio-demographics shape KAP outcomes, a perspective often missing in global summaries.

However, several limitations temper the interpretation of these findings. First, the cross-sectional design precludes causal conclusions about the drivers of disposal behaviors. Second, the use of non-probabilistic snowball sampling via social media led to a pronounced demographic skew, resulting in an unusually well-educated sample (81.8% holding a university degree) that was predominantly young (mean age 27.2 years) and female. This likely resulted in an overestimation of actual knowledge and motivation compared to the general population, where older or less-educated individuals were underrepresented. Furthermore, our sampling was confined to the West Bank and may not fully capture Gaza or refugee populations, limiting generalizability. These demographic imbalances also made subgroup analyses exploratory and underpowered; *p*-values were not adjusted for multiple comparisons, so the possibility of Type I error cannot be excluded. Third, some limitations are inherent to the instrument; the knowledge scale’s internal consistency was lower than the attitude scale, suggesting a need for future refinement, and the online format prevented probing for misunderstandings on potentially confusing questions (e.g., inhaler disposal acceptability). Finally, all data were self-reported, which introduces potential recall and social desirability biases regarding unsafe disposal practices; collectively, these findings should be viewed as preliminary and interpreted with caution.

### Novel contributions and broader significance

This study makes several important contributions to the global literature on pharmaceutical waste management in resource-limited and fragile healthcare systems. First, it provides the first population-level assessment of medication disposal KAP in Palestine, addressing a critical evidence gap in a conflict-affected setting where informal waste practices may exacerbate both environmental contamination and antimicrobial resistance. While previous studies in the region have examined healthcare workers’ practices (9), our focus on the general public reveals the extent of unsafe household disposal behaviors that contribute to pharmaceutical pollution in settings without formal waste management infrastructure. Second, our application of the Health Belief Model with causal mediation analysis advances methodological understanding by demonstrating that perceived risk and structural barriers operate as independent, parallel determinants rather than through a causal chain (ACME *p* = 0.896). This finding has critical policy implications: it empirically demonstrates that educational campaigns focused solely on increasing awareness—the most common intervention in low-resource settings—will have limited impact if structural barriers remain unaddressed. This evidence-based insight challenges the predominant reliance on awareness-raising in resource-constrained contexts and provides quantitative justification for dual-pronged strategies. Third, our findings directly inform antimicrobial resistance prevention efforts. The fact that only 29.4% of participants recognized the link between improper antibiotic disposal and AMR, combined with widespread garbage disposal practices (77%), highlights a critical vulnerability in settings where environmental surveillance is absent and antibiotic use is high. Antibiotics entering municipal waste and water systems in such contexts create selection pressure for resistant bacteria, contributing to the global AMR crisis. Finally, our phased, resource-appropriate implementation framework provides a practical model adaptable to similar fragile and low- to middle-income settings globally, offering a roadmap that prioritizes low-cost, high-impact interventions (pharmacy-based collection, brief training, social media campaigns) before capital-intensive solutions (specialized incineration facilities, comprehensive waste treatment infrastructure). This approach is particularly relevant for the fragile and conflict-affected states identified by the World Bank, many of which face similar challenges of limited health infrastructure, growing pharmaceutical consumption, and rising AMR.

### Future research

Future studies are needed to build on these preliminary findings. Larger-scale surveys using probability sampling across diverse Palestinian communities would help confirm whether these patterns hold broadly. Longitudinal or experimental research designs could establish causality and test the impact of specific interventions on disposal behavior. The survey instrument itself could be refined to better distinguish factual knowledge from beliefs and to measure actual disposal frequency more directly. Qualitative research (e.g., interviews or focus groups) would also be valuable to uncover in-depth reasons behind disposal choices and perceived barriers. Environmental monitoring (e.g., measuring pharmaceutical residues in water) would also complement KAP studies and quantify the impact of disposal initiatives. Importantly, pilot evaluations of proposed interventions (such as disposal kiosks, educational programs, or incentive schemes) should accompany future research. These evaluations would identify which approaches are most acceptable and effective in this setting. Together, a multi-method research agenda can generate robust evidence to inform tailored public health strategies in Palestine.

## Conclusion

This study provides critical evidence on medication disposal practices in a fragile, resource-limited healthcare system, revealing that unsafe disposal behaviors are pervasive despite positive attitudes and general awareness of environmental risks. The findings demonstrate that structural barriers operate independently of risk perception, providing empirical justification for dual-pronged interventions that combine education with accessible infrastructure—a model essential for effective pharmaceutical waste management and antimicrobial resistance prevention in similar settings globally. Given the cross-sectional design and sampling bias, these results are preliminary and should be interpreted cautiously. Nonetheless, our findings underscore the immediate necessity of developing a national strategy that integrates low-cost, pharmacy-based take-back programs with sustained public education campaigns. The proposed phased implementation framework, grounded in Health Belief Model principles and adapted to Palestinian healthcare system constraints, offers a scalable model for fragile and conflict-affected states and numerous low- and middle-income countries facing concurrent challenges of inadequate waste infrastructure and rising antimicrobial resistance. Successful mitigation of environmental contamination, pharmaceutical pollution of water systems, and AMR selection pressure requires both policy reform and public engagement to establish formal disposal systems. These findings contribute to the limited but growing evidence base on household pharmaceutical waste management in vulnerable settings and provide an actionable roadmap for policymakers, public health authorities, and international development partners committed to sustainable health system strengthening and global AMR containment.

## Data Availability

The raw data supporting the conclusions of this article will be made available by the authors, without undue reservation.
