# Supplementary material for: Public knowledge and practices toward safe disposal of unused and expired medications in Palestine: a cross-sectional study
Source: Front Public Health. 2026 Sep 1;14:1944632. doi: 10.3389/fpubh.2026.1944632 (PMC13574934; doi:10.3389/fpubh.2026.1944632)
Supplement: Supplementary file 1 [file Table_1.DOCX]

**Supplementary Material legends:**

Supplementary 1. Differences in participants’ knowledge by gender (N = 402).

Supplementary 2. Differences in participants’ attitudes by gender (median and mean).

Supplementary 3. Association between education level and knowledge (N = 402).

Supplementary 4. Differences in participants’ knowledge according to age (N = 402).

Supplementary 5. Association between income and knowledge (N = 402).

**Supplementary Material:**

**Supplementary 1. Differences in participants’ knowledge by gender (N = 402).**

| **Knowledge Item** | **Gender** | **Correct (n, %)** | **Incorrect (n, %)** | **p** |
| --- | --- | --- | --- | --- |
| **Improper disposal of expired or unused medications has harmful effects on the environment** | Male | 89 (67.9%) | 42 (32.1%) | 0.086 |
|  | Female | 206 (76.0%) | 65 (24.0%) |  |
| **Medications reach groundwater if thrown in the toilet/sink** | Male | 54 (41.2%) | 77 (58.8%) | 0.904 |
|  | Female | 110 (40.6%) | 161 (59.4%) |  |
| **Acceptability of disposing solid medications (pills/capsules) in garbage** | Male | 52 (39.7%) | 79 (60.3%) | **0.042*** |
|  | Female | 80 (29.5%) | 191 (70.5%) |  |
| **Improper disposal of antibiotics leads to resistance** | Male | 40 (30.5%) | 91 (69.5%) | 0.718 |
|  | Female | 78 (28.8%) | 193 (71.2%) |  |
| **Burning is the environmentally sound way to dispose of unwanted medications** | Male | 23 (17.6%) | 108 (82.4%) | 0.116 |
|  | Female | 32 (11.8%) | 239 (88.2%) |  |
| **It is acceptable to dispose of needles and syringes in the garbage** | Male | 66 (50.4%) | 65 (49.6%) | 0.467 |
|  | Female | 147 (54.2%) | 124 (45.8%) |  |
| **Acceptability of disposing inhalers (asthma sprays) in garbage** | Male | 41 (31.3%) | 90 (68.7%) | **0.038*** |
|  | Female | 114 (42.1%) | 157 (57.9%) |  |
| **It is acceptable to dispose of creams and ointments in the garbage** | Male | 31 (23.7%) | 100 (76.3%) | 0.532 |
|  | Female | 72 (26.6%) | 199 (73.4%) |  |

*Note: p < 0.05 indicates a statistically significant gender difference.

**Supplementary 2. Differences in participants’ attitudes by gender (Mann–Whitney U test)**

| **Attitude Item** | **Gender** | **N** | **Median** | **IQR** | **Mean** | **SD** | **p** |
| --- | --- | --- | --- | --- | --- | --- | --- |
| **It is my responsibility to protect my family members from harmful medications** | Male | 131 | 4 | 1 | 4.27 | 0.869 | 0.210 |
|  | Female | 271 | 4 | 1 | 4.16 | 0.915 |  |
| **I am willing to donate my unused medications before they expire** | Male | 131 | 4 | 1 | 3.98 | 1.019 | 0.758 |
|  | Female | 271 | 4 | 1 | 3.99 | 1.042 |  |
| **If there was a financial incentive for me to return unused medications** | Male | 131 | 4 | 2 | 3.89 | 0.908 | 0.113 |
|  | Female | 271 | 4 | 1 | 3.70 | 1.031 |  |
| **Doctors, pharmacists, and health professionals should provide advice on disposal** | Male | 131 | 5 | 1 | 4.36 | 0.775 | 0.072 |
|  | Female | 271 | 5 | 1 | 4.49 | 0.740 |  |
| **Awareness programs should be initiated on how to dispose of medications** | Male | 131 | 4 | 1 | 4.18 | 0.949 | **0.003*** |
|  | Female | 271 | 5 | 1 | 4.45 | 0.733 |  |
| **There should be public awareness programs about disposal effects** | Male | 131 | 4 | 1 | 4.18 | 0.830 | **<0.001*** |
|  | Female | 271 | 5 | 1 | 4.47 | 0.719 |  |
| **Community pharmacists should participate in awareness about disposal** | Male | 131 | 4 | 2 | 3.89 | 1.017 | 0.770 |
|  | Female | 271 | 4 | 2 | 3.92 | 1.033 |  |
| **There is a lack of sufficient information about safe disposal** | Male | 131 | 4 | 1 | 4.07 | 0.896 | **0.012*** |
|  | Female | 271 | 4 | 1 | 4.28 | 0.827 |  |
| **Children are more exposed to risks associated with unused/expired medications** | Male | 131 | 4 | 1 | 4.11 | 0.905 | 0.145 |
|  | Female | 271 | 4 | 1 | 4.25 | 0.797 |  |
| **It is convenient for me to take my unused or expired medications to the pharmacyfor proper disposal** | Male | 131 | 4 | 1 | 3.56 | 1.075 | 0.795 |
|  | Female | 271 | 4 | 1 | 3.53 | 1.141 |  |

*Note: p < 0.05 indicates a statistically significant gender difference; significant items are italicized.

**Supplementary 3. Association between education level and knowledge (N=402)**

| **Knowledge Item** | **Secondary or less (n=34) N / Total (%)** | **University (n=329) N / Total (%)** | **Postgraduate (n=39) N / Total (%)** | **p-value*** |
| --- | --- | --- | --- | --- |
| **Improper disposal harms the environment.** | 21 / 34 (61.8%) | 250 / 329 (76.0%) | 24 / 39 (61.5%) | **0.043*** |
| **Medications reach groundwater if flushed.** | 12 / 34 (35.3%) | 136 / 329 (41.3%) | 16 / 39 (41.0%) | 0.792 |
| **Disposing solid medications (pills/capsules) in garbage is acceptable.** | 15 / 34 (44.1%) | 108 / 329 (32.8%) | 9 / 39 (23.1%) | 0.162 |
| **Improper disposal of antibiotics leads to resistance.** | 7 / 34 (20.6%) | 95 / 329 (28.9%) | 16 / 39 (41.0%) | 0.145 |
| **Burning is an environmentally sound disposal method.** | 6 / 34 (17.6%) | 41 / 329 (12.5%) | 8 / 39 (20.5%) | 0.300 |
| **Disposing needles and syringes in garbage is acceptable.** | 17 / 34 (50.0%) | 176 / 329 (53.5%) | 20 / 39 (51.3%) | 0.904 |
| **Disposing inhalers in garbage is acceptable.** | 18 / 34 (52.9%) | 121 / 329 (36.8%) | 16 / 39 (41.0%) | 0.173 |
| **Disposing creams and ointments in garbage is acceptable.** | 10 / 34 (29.4%) | 82 / 329 (24.9%) | 11 / 39 (28.2%) | 0.788 |

Note: p < 0.05 indicates a statistically significant association.

**Supplementary 4. Differences in participants’ knowledge according to age (N = 402).**

| **Knowledge Item** | **Correct (N)** | **Mean age (SD)** | **Incorrect (N)** | **Mean age (SD)** | **p** |
| --- | --- | --- | --- | --- | --- |
| **Improper disposal harms the environment** | 295 | 27.4 (9.95) | 107 (26.8) |  | 0.601 |
| **Medications reach groundwater if flushed** | 164 | 27.0 (9.41) | 238 (27.4) | 27.4 | 0.668 |
| **Acceptability of disposing solid medications in the garbage** | 132 | 25.4 (8.96) | 270 (28.1) | 28.1 | **0.008*** |
| **Improper disposal of antibiotics leads to resistance** | 118 | 26.8 (9.88) | 284 (27.4) | 27.4 | 0.625 |
| **Burning is an environmentally sound way to dispose of unwanted medications** | 55 | 28.9 (11.0) | 347 (26.9) | 26.9 | 0.164 |
| **Acceptability of disposing needles and syringes in the garbage** | 213 | 26.1 (9.05) | 189 (28.4) | 28.4 | **0.016*** |
| **Disposing inhalers in the garbage is acceptable** | 155 | 26.9 (9.81) | 247 (27.4) | 27.4 | 0.611 |
| **Disposing creams/ointments in the garbage is acceptable** | 103 | 27.5 (10.4) | 299 (27.1) | 27.1 | 0.729 |

*Note: p < 0.05 indicates a statistically significant age difference.

**Supplementary 5. Association between income and knowledge (N = 402).**

| **Knowledge Item** | **Low income (n=122)**  **% Correct** | **Average income (n=244)**  **% Correct** | **High income (n=36)**  **% Correct** | **p** |
| --- | --- | --- | --- | --- |
| **Improper disposal harms the environment** | 67.2% | 77.5% | 66.7% | 0.071 |
| **Medications reach groundwater if thrown in the toilet/sink** | 33.6% | 41.8% | 58.3% | **0.026*** |
| **Acceptability of disposing solid medications in garbage** | 35.2% | 31.6% | 33.3% | 0.776 |
| **Improper disposal of antibiotics leads to resistance** | 28.7% | 27.9% | 41.7% | 0.233 |
| **Burning is an environmentally sound disposal method** | 10.7% | 14.3% | 19.4% | 0.359 |
| **Acceptability of disposing needles/syringes in the garbage** | 50.8% | 54.1% | 52.8% | 0.839 |
| **Acceptability of disposing inhalers in garbage** | 36.1% | 39.3% | 41.7% | 0.767 |
| **Acceptability of disposing creams/ointments in garbage** | 27.9% | 25.0% | 22.2% | 0.744 |

*Note: p < 0.05 indicates a statistically significant association.
